# Low-grade postoperative spine infection with a history of oral cavity infections: think of *Peptoniphilus asaccharolyticus*

**DOI:** 10.1093/jscr/rjae625

**Published:** 2024-11-08

**Authors:** Konstantinos Zygogiannis, Eleni Pappa, Spiridon Antonopoulos, Ioannis Chatzikomninos, Anastasios Kalampokis

**Affiliations:** Spine Surgery and Scoliosis Department, KAT General Hospital, Nikis 2 Street, Kifisia, Athens 14561, Greece; Spine Surgery and Scoliosis Department, KAT General Hospital, Nikis 2 Street, Kifisia, Athens 14561, Greece; Spine Surgery and Scoliosis Department, KAT General Hospital, Nikis 2 Street, Kifisia, Athens 14561, Greece; Spine Surgery and Scoliosis Department, KAT General Hospital, Nikis 2 Street, Kifisia, Athens 14561, Greece; Spine Surgery and Scoliosis Department, KAT General Hospital, Nikis 2 Street, Kifisia, Athens 14561, Greece

**Keywords:** spine infection, low grade infection, *Peptoniphilus asaccharolyticus*

## Abstract

*Peptoniphilus asaccharolyticus* is a typical gram-positive commercial microorganism of the skin that depending on the occasion can also be detected in the gut and in the genitourinary system. There is a paucity in the literature regarding the role of *P. asaccharolyticus* in spine infections and the potential impact in postoperative implications. A case report of a patient suffering from infection of spinal instrumentation of the uncommon pathogen above is presented.

## Introduction

Gram-positive anaerobic cocci can usually be found in polymicrobial infections, especially in patients with long periods of hospitalization and less in monomicrobial infections. *Peptoniphilus asaccharolyticus* is a typical gram-positive commensal microorganism of the skin that depending on the occasion can also be detected in the gut and in the genitourinary system [1]. The most common course of its action is not opportunistic, meaning its prevalence is higher in immunosuppressed patients with chronic skin ulcer, chronic oral cavity infections and wounds in a selected group of patients such as diabetics [2]. There is a paucity in the literature regarding the role of *P. asaccharolyticus* in spine infections and the potential impact in postoperative implications.

Postoperative spine infection can be divided in early onset or low grade. They are often demanding in diagnosis and treatment as early signs immediately postoperatively may often slip. Within the site of infection, areas such as the epidural space, the spinal canal, the surrounding soft tissues, and bone may also be affected causing abscess or even osteomyelitis [3]. The incidence of postoperative spine infection is multifactorial and may have a wide range of variation from 0 to 18% depending on the patient’s comorbidities, history of infections, or trauma [4]. Our study aims to raise awareness of low-grade spine infections secondary to oral cavity infections caused by *P. asaccharolyticus*.

## Case presentation

**Figure 1:**
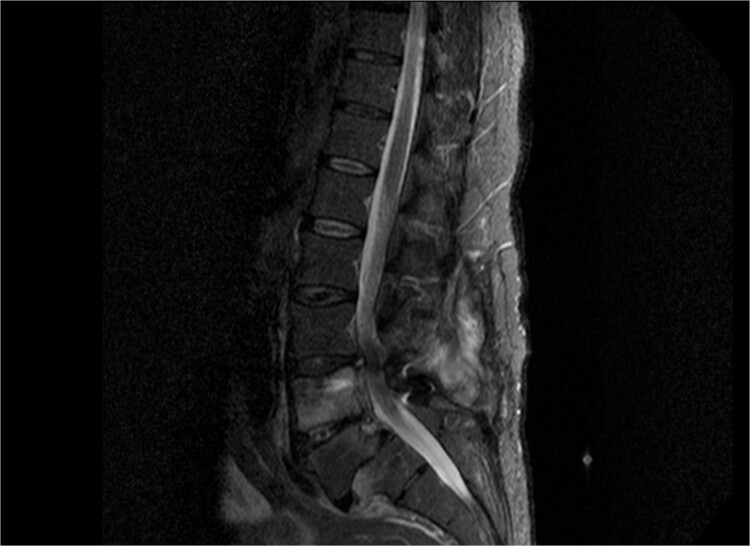
Sagittal view of MRI of lumbar spine demonstrating diffuse soft tissue pathological sign, with signs of osteomyelitis at the level of L4 and L5.

**Figure 2:**
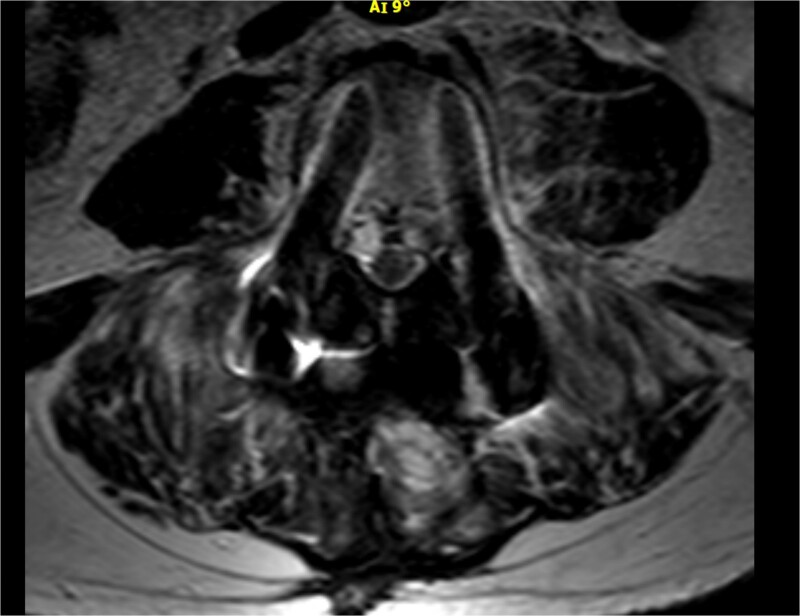
Axial view of the MRI scan demonstrating pathological fluid collection around the pedicle screws.

A 64 year-old patient with a history of type I diabetes underwent posterior lumbar fixation with decompression for spinal canal stenosis in November 2023, in our hospital. The patient followed a normal postoperative period with clinical improvement of neurological claudication and back pain based on ODI scores. In May 2024, the patient presented with acute sudden onset debilitating back pain and fever, without any previous symptomatology regarding lumbar spine. From the patient’s history, since the end of January the patient was suffering from oral cavity infection with multiple recurrences of abscesses. The first round of blood examinations revealed the following results:

As shown in Table 1, the patient’s blood tests were compatible with infection and an MRI-CT scan was ordered to confirm or exclude the participation of lumbar spine. Subsequently, the MRI revealed signs compatible with the diagnosis of osteomyelitis involving the L4 and L5 vertebra as shown in sagittal view in Fig. 1 where screws were placed, with the presence of pathological collection of fluid around the pedicle screws and diffuse soft tissue edema as shown in the axial view in Fig. 2. Additionally, the CT scan revealed septic loosening of the pedicle screws. Immediately the patient underwent surgical irrigation and debridement with placement of new larger pedicle screws due to the absence of union. Intraoperatively eight different samples were collected for culture from the surrounding soft tissue and bone, while the previous pedicle screws were sent for sonication. Postoperatively all the blood cultures were negative along with the soft tissue and bone, only the sonication revealed infection with *P. asaccharolyticus*

**Table 1:** First round of patient’s blood examination.

Examination	Value (normal value)
WBC	10.100 (4.6–10.2 × 10^3^/μL)
CRP	28.90 (<0.31 mg/dL)
Procalcitonin	4.01 (<0.50 ng/mL)
Erythrocyte sed rate	56 (0–20 mm)
Blood cultures	Negative
Neutrophils	76.40 (40–74%)

## Discussion

The incidence of postoperative spinal infections ranges from 0.7 to 12% depending on various factors and studies such as the type of surgery, patient comorbidities, and perioperative protocols. Schuster *et al.* in a systematic review suggested that the incidence was found to be ~4% in spinal surgeries [5]. Major risk factors include diabetes mellitus, obesity, smoking, prolonged operative time, and previous spine surgery [6]. For example, a study made by Pull ter Gunne and Cohen reported that diabetes increased the risk of postoperative spinal infections by 2.3 times [7]. The most common causative agents of spinal infections are *Staphylococcus aureus* and coagulase-negative Staphylococci, which account for ~50 to 70% of cases. *Cutibacterium acnes* is also a notable pathogen, particularly in late postoperative infections [8]. Mortality rates associated with spinal infections vary, but studies have reported rates from 2 to 17% [9]. Additionally, a review by Mackenzie *et al.* indicated a mortality rate of ~5% in patients with spinal infections [10]. Recurrence rates of spinal infections after treatment range from 10 to 30%, with higher rates observed in cases where instrumentation is retained. Di Silvestre *et al.* found a 50% probability of persistent infection when instrumentation was retained during the debridement of delayed spinal infections [11]. Τhe species of *P. asaccharolyticus* has also been mentioned in the current literature as a cause factor of delayed infection also in total hip arthroplasty, as it was grown from the sonication of the implants, so not only the spine surgeons but also general orthopedic surgeons should also be aware of the management of the microbe above [12]. Also, *P. asaccharolyticus* can also cause secondary infection in paraspinal abscesses, as stated by Sreenivasan *et al.*, as in their case report of a diabetic also patient, anaerobic culture of the paraspinal pus revealed the microbe above co-existing with the *Mycobacterium Tuberculosis*. [13] Future directions and recommendations should include a thorough patient’s medical history and detailed information of the importance and impact of infections in patients with medical implants.

## Conclusion

Low-grade postoperative infections can potentially have a devastating impact on clinical surgical outcomes as they may remain silent at the very start until they produce pathologies such as osteomyelitis and septic loosening of implants. Doctor–patient communication should be promoted even in cases where the infection is irrelevant with the lumbar spine.
